# Identification of a CO_2_ Responsive Regulon in *Bordetella*

**DOI:** 10.1371/journal.pone.0047635

**Published:** 2012-10-24

**Authors:** Sara E. Hester, Minghsun Lui, Tracy Nicholson, Daryl Nowacki, Eric T. Harvill

**Affiliations:** 1 Department of Veterinary and Biomedical Sciences, The Pennsylvania State University, University Park, Pennsylvania, United States of America; 2 Graduate Program in Biochemistry, Microbiology, and Molecular Biology, The Pennsylvania State University, University Park, Pennsylvania, United States of America; 3 Department of Microbiology, Immunology, and Molecular Genetics, David Geffen School of Medicine at UCLA, University of California Los Angeles, Los Angeles, California, United States of America; 4 National Animal Disease Center, Agricultural Research Service, United States Department of Agriculture, Ames, Iowa, United State of America; University of Illinois at Chicago College of Medicine, United States of America

## Abstract

Sensing the environment allows pathogenic bacteria to coordinately regulate gene expression to maximize survival within or outside of a host. Here we show that *Bordetella* species regulate virulence factor expression in response to carbon dioxide levels that mimic *in vivo* conditions within the respiratory tract. We found strains of *Bordetella bronchiseptica* that did not produce adenylate cyclase toxin (ACT) when grown in liquid or solid media with ambient air aeration, but produced ACT and additional antigens when grown in air supplemented to 5% CO_2_. Transcriptome analysis and quantitative real time-PCR analysis revealed that strain 761, as well as strain RB50, increased transcription of genes encoding ACT, filamentous hemagglutinin (FHA), pertactin, fimbriae and the type III secretion system in 5% CO_2_ conditions, relative to ambient air. Furthermore, transcription of *cyaA* and *fhaB* in response to 5% CO_2_ was increased even in the absence of BvgS. *In vitro* analysis also revealed increases in cytotoxicity and adherence when strains were grown in 5% CO_2_. The human pathogens *B. pertussis* and *B. parapertussis* also increased transcription of several virulence factors when grown in 5% CO_2_, indicating that this response is conserved among the classical bordetellae. Together, our data indicate that *Bordetella* species can sense and respond to physiologically relevant changes in CO_2_ concentrations by regulating virulence factors important for colonization, persistence and evasion of the host immune response.

## Introduction

Many cues, such as temperature, oxygen (O_2_), iron, pH, osmolarity and bicarbonate, allow bacteria to distinguish between environments within a host and outside of a host, as well as various microenvironments within a host [1]. In sensing multiple cues, bacteria are able to synchronize gene expression to adapt and ultimately thrive [2]. One cue, carbon dioxide (CO_2_), has been shown to affect regulation of virulence factor expression in many bacterial pathogens. *Bacillus anthracis* responds to elevated levels of CO_2_ by increasing expression of the genes encoding edema toxin, lethal factor and protective antigen [3]–[5]. In response to 10% CO_2,_ *Streptococcus pyogenes* increases transcription of M protein, an important virulence factor that prevents the deposition of complement onto the bacterial surface [6]. In increased CO_2_, M protein has been shown to be regulated by a trans-acting positive regulatory protein that binds to the promoter of the *emm* gene [6], [7]. CO_2_ regulation in *B. anthracis* appears to be more complicated since the transcriptional regulator of the toxins is not increased transcriptionally in response to growth in CO_2_ [3]. Additionally, *Staphylococcus aureus, Salmonella enterocolitica* and *Borrelia burgdorferri* are responsive to increased CO_2_ concentrations, suggesting this ability is useful to a variety of pathogens [8]–[11].

*Bordetella bronchiseptica* is a Gram-negative bacterium that infects a wide range of hosts causing respiratory disease varying from asymptomatic persistence in the nasal cavity for the life of the host to lethal pneumonia [12]–[14]. *B. bronchiseptica* is very closely related to the other two classical bordetellae, *Bordetella pertussis* and *Bordetella parapertussis*, the causative agents of whooping cough in humans [13], [15], [16]. Several virulence factors are produced by *B. bronchiseptica* such as, pertactin (PRN), filamentious hemaglutinin (FHA), two serotypes of fimbriae, and the two cytotoxic mechanisms, adenylate cyclase toxin (ACT) and the Type III Secretion System (TTSS) [13], [16]. ACT, a member of the repeats-in-toxin (RTX) family, is a bi-functional adenylate cyclase/hemolysin that converts ATP to cAMP, disrupting oxidative burst, phagocytosis, chemotaxis and eventually leads to apoptosis in macrophages and neutrophils [17]–[19]. ACT has also been shown to contribute to pathology, efficient colonization and persistence of *B. bronchiseptica* and *B. pertussis* species [20]–[22].

Regulation of virulence factors in bordetellae occurs via the BvgAS two-component system [23]. BvgS, the sensor in the cytoplasmic membrane, is thought to directly sense changes in the environment and, through a phosphorylation-transfer mechanism, activates BvgA, the response regulator [24]–[26]. Once BvgA is activated (Bvg^+^ phase), it binds to high and low affinity motifs in the genome, resulting in increased expression of the genes encoding toxins and adhesins, while expression of Bvg^−^ phase genes involved in motility and uptake of certain nutrients are repressed; the opposite occurs in the Bvg^−^ phase [27]–[32]. An intermediate phase has been described in which a subset of virulence factors are expressed, along with a unique set of factors [33]–[35]; however the Bvg^+^ phase has been shown to be necessary and sufficient for host colonization [36]. Although BvgAS appears to be sufficient for regulation of virulence factors the ability to respond to multiple signal inputs to differentially regulate transcriptional networks likely allows for adaptation to different microenvironments within the host. *Bordetella* species have multiple putative transcription factors within their genomes, indicating that gene regulation is likely to be a more complex regulatory system than is currently appreciated [16].

Here we identify, through screening of a collection of *B. bronchiseptica* isolates, strains that only produce ACT in response to growth in elevated CO_2_ conditions. Both strain 761 and the sequenced laboratory reference strain RB50 increased transcription of *cyaA* and production of ACT when grown in 5% CO_2_ conditions, although only strain 761 was dependent on 5% CO_2_ for efficient expression. Several other virulence factor genes were increased in transcription in response to growth in elevated CO_2_. BvgAS was required for ACT production, but *cyaA* and *fhaB* were transcriptionally increased in response to 5% CO_2_ conditions in the absence of BvgS. Together this indicates that an additional regulatory system increases production of ACT and other virulence factors in various *Bordetella* species.

## Materials and Methods

### Ethics Statement

This study was carried out in strict accordance with the recommendations in the Guide for the Care and Use of Laboratory Animals of the National Institutes of Health. The protocol was approved by the Institutional Animal Care and Use Committee at The Pennsylvania State University at University Park, PA (#31297 Bordetella-Host Interactions). All animals were anesthetized using isoflourane or euthanized using carbon dioxide inhalation to minimize animal suffering.

**Figure 1:**
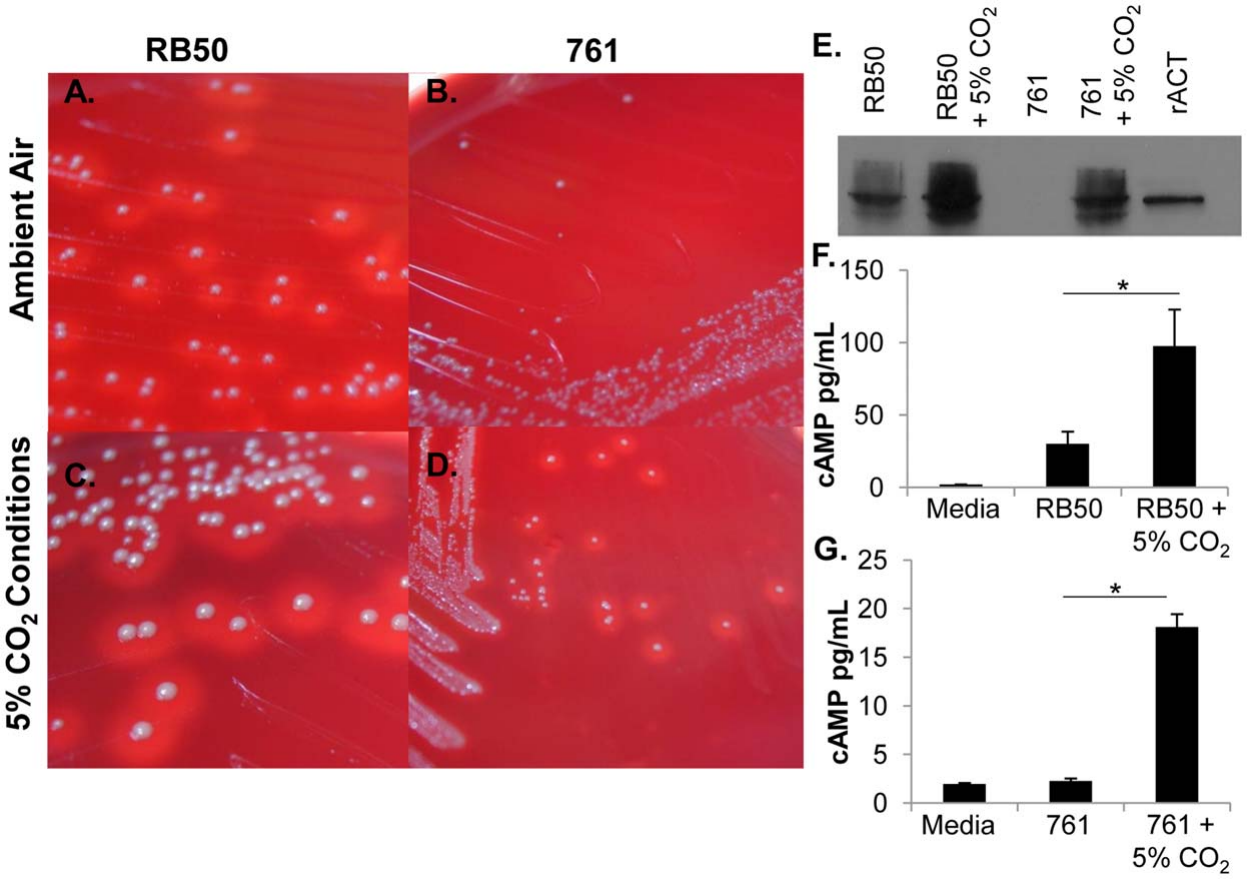
Differential production of ACT in *B. bronchiseptica* strains grown in 5% CO_2_. Strains RB50 (A, C) and 761 (B, D), grown in normal atmospheric oxygen conditions (A,B) or in elevated 5% CO_2_ conditions (C, D). (E) RB50 and 761 grown in atmospheric conditions or grown in 5% CO_2_ conditions, or recombinant ACT (2.5 ng) were probed with a monoclonal antibody to CyaA protein at a dilution of 1∶1000. J774 murine macrophage cells were stimulated with media or media containing RB50 (F) or 761 (G) at an MOI of 1 for 30 minutes, and cAMP levels were assessed. * indicates a p-value less than 0.05.

### Bacterial Strains and Growth

*B. bronchiseptica* strain RB50 is an isolate from a rabbit [36]. RB54 and RB50Δ*bscN*Δ*cyaA* are previously described derivatives of strain RB50 [33], [37]. *B. bronchiseptica* strain 761, 448, and 308 were obtained from the CDC in Atlanta, Georgia and have been previously described [15], [38], [39]. *B. bronchiseptica* strain JC100 has been previously described [15]. *B. parapertussis* strain 12822 was isolated from German clinical trials and has been previously described [40], [41]. *B. pertussis* strain 536 is a streptomycin resistant derivative of Tohama I [42] and strain 18323 has been previously described [43]. *B. pertussis* strain CHOC 0012 was isolated on Regan-Lowe media by the Eunice Kennedy National Insitute of Child Health and Human Development (NICHD) Collaborative Pediatric Critical Care Research Network from a child displaying severe Pertussis. Bacteria were maintained on Bordet-Gengou agar (Difco, Sparks, MD) containing 10% sheep blood (Hema Resources, Aurora OR) and 20 µg/mL streptomycin (Sigma Aldrich, St. Louis, MO). Liquid cultures were grown at 37°C overnight in a shaker to mid-log phase (O.D. 0.7–1.0) in Stainer-Scholte (SS) broth. Bacteria were grown overnight with constant shaking (250 rpm) in standard glass test tubes in either atmospheric concentrations of oxygen and carbon dioxide (atmospheric conditions) or in atmospheric levels of oxygen with the constant controlled addition of 5% carbon dioxide into a sealed incubator 37°C (5% CO_2_ conditions).

**Figure 2:**
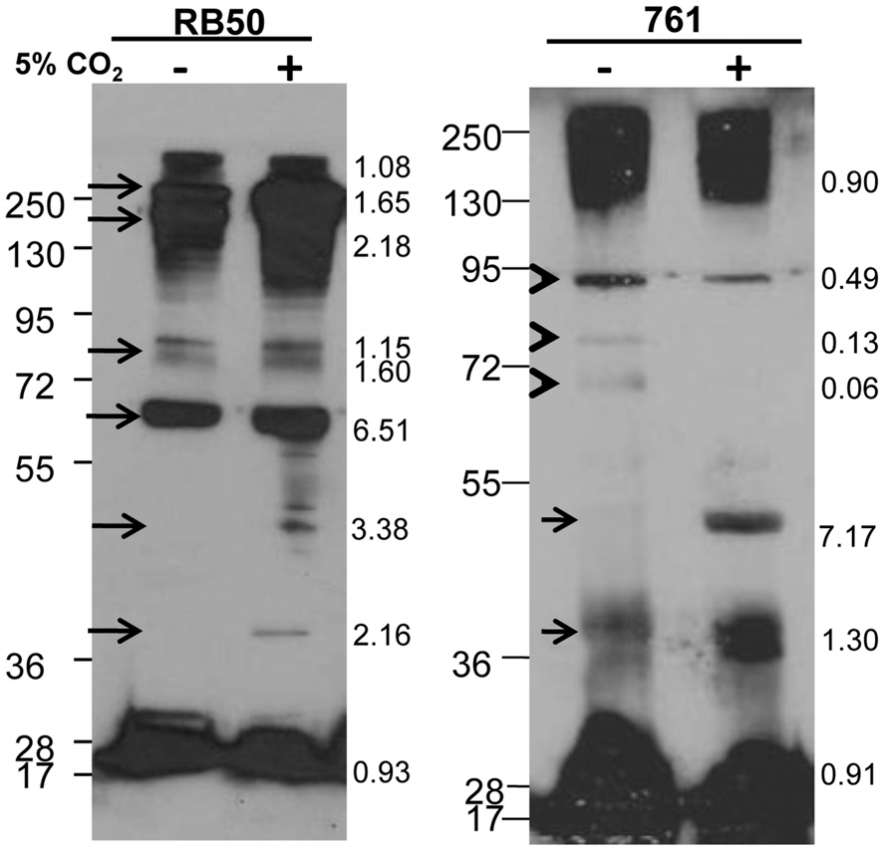
Differential recognition of antigens in *B. bronchiseptica* strains grown in atmospheric or 5% CO_2_ conditions. C57BL/6 mice were inoculated with 5×10^5^ CFU *B. bronchiseptica* strain RB50, and serum was collected 28 days later. Strains RB50 and 761 were grown in atmospheric or in elevated CO_2_ concentrations, and were probed with serum against RB50. Increased (arrows) or decreased (arrowheads) production of antigens in response to 5% CO_2_ conditions is denoted. The ratio of band intensity between antigens produced in ambient air and 5% CO_2_ conditions is indicated in the margins: 1 = equal amounts produced in either conditions, <1 = more produced in ambient air, >1 = more produced in 5% CO_2_ conditions.

### cAMP Assay

Murine macrophage-like cell line, J774, was cultured in Dulbecco modified Eagle medium (DMEM) with 10% fetal bovine serum (FBS) (Hyclone Laboratories, Inc., Logan, UT). Cells were grown to approximately 80% confluency, and bacteria were added at a multiplicity of infection (MOI) of 1. After a 5 minute centrifugation at 250×*g*, the mixture was incubated for 30 minutes at 37°C. cAMP was measured with a cyclic AMP ELISA system (Tropix, Bedford, MA) according to the manufacturer’s instructions. Results were analyzed using analysis of variance with a Tukey simultaneous test, and a *P* value of <0.05 was considered significant.

**Figure 3:**
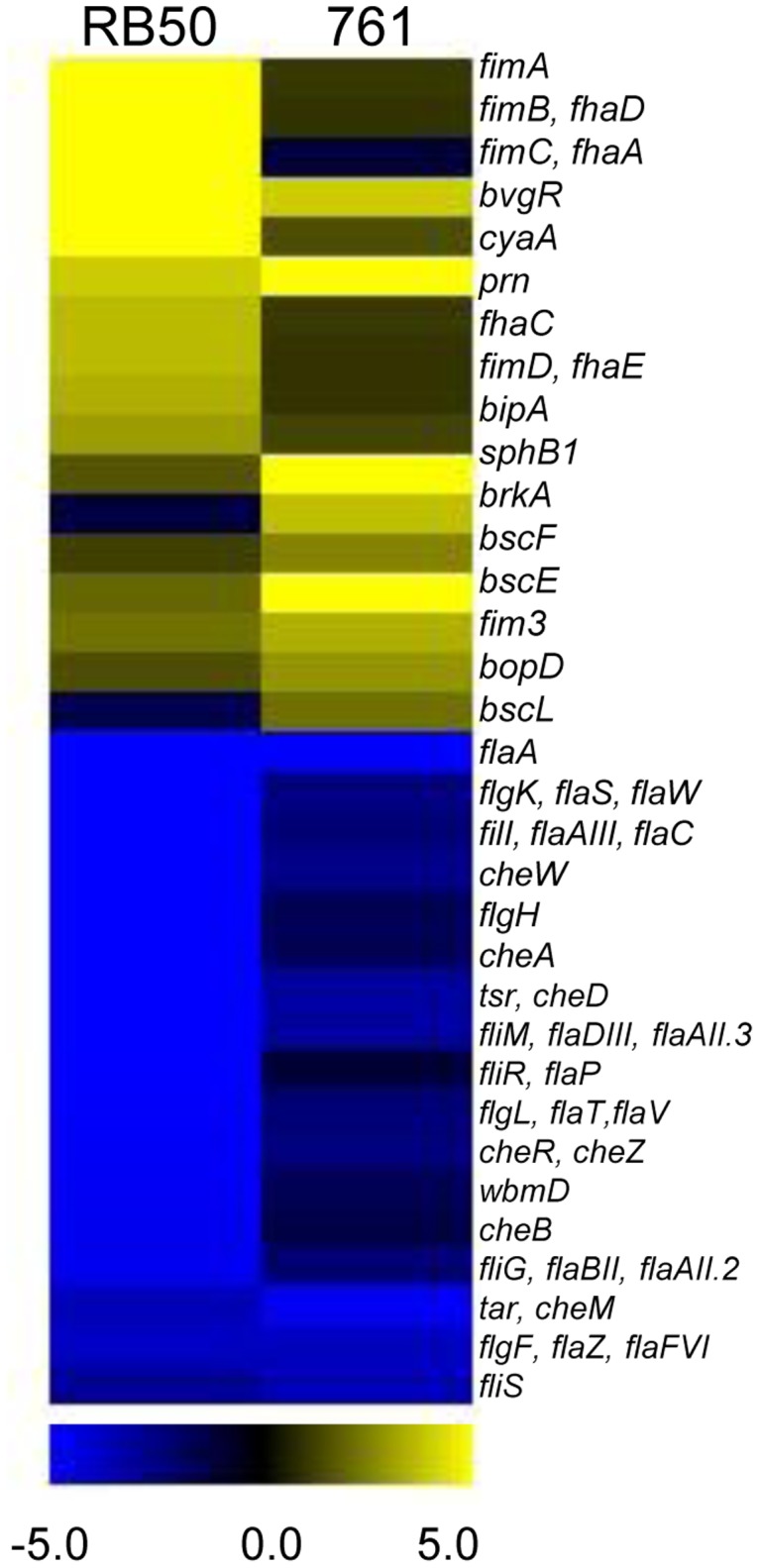
Defining the CO_2_ responsive regulon in *B. bronchiseptica*. Changes in gene expression of *B. bronchiseptica* in response to 5% CO_2_ are analyzed by MeV analysis [48]. Several known virulence factor genes reported to be regulated by BvgAS in prototypical *B. bronchiseptica* strain RB50 are shown for strain RB50 (left) and strain 761 (right), with yellow representing increased transcription and blue indicative of decreased transcription in growth in 5% CO_2_ conditions, compared to growth in normal atmospheric conditions.

**Figure 4:**
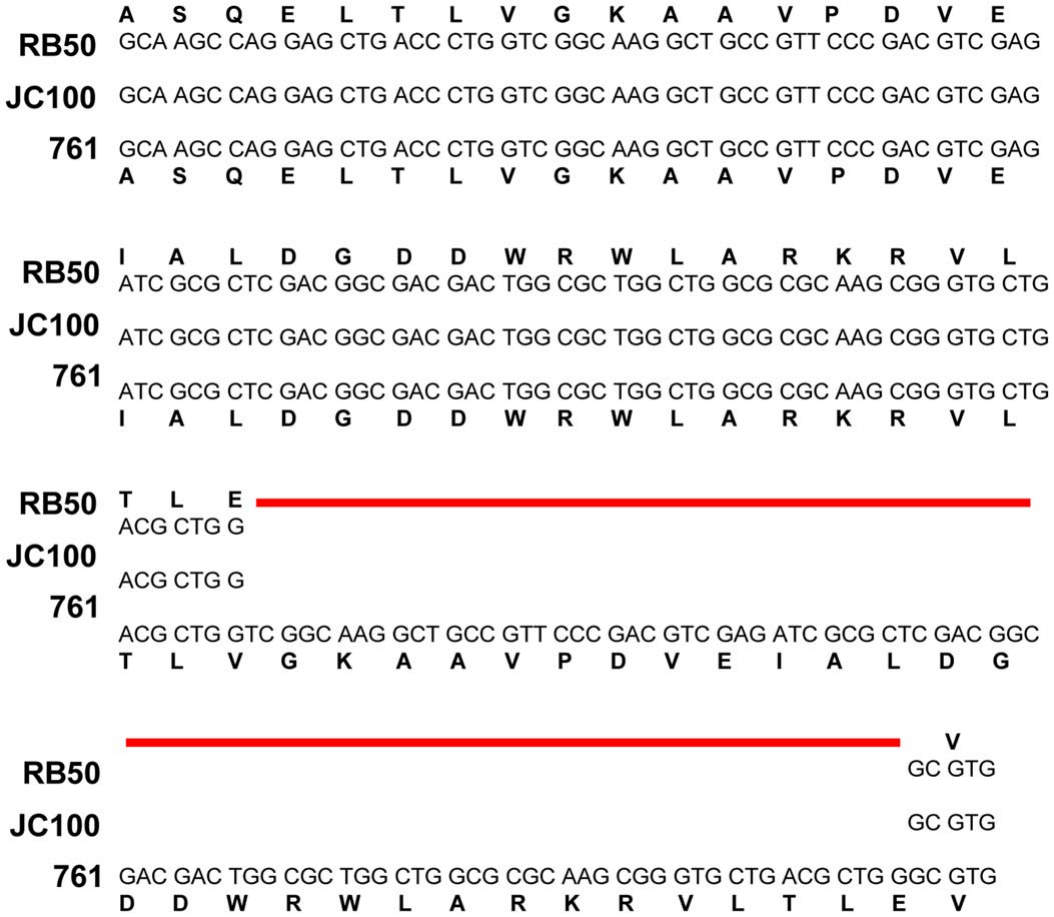
Duplication in the *bvgS* gene in strain JC100. The DNA and protein sequences of *bvgS* gene from JC100 are aligned against those of *bvgS* gene from RB50 and 761. The red line indicates the 29 amino acid duplication in the *bvgS* locus in JC100 compared to RB50 and 761.

### Animal Experiments

C57BL/6 mice were obtained from Jackson Laboratories (Bar Harbor, ME). Mice were bred in our *Bordetella*-free, specific pathogen-free breeding rooms at The Pennsylvania State University. All animal experiments were performed in accordance with institutional animal care and use committee (IACUC) guidelines. 4 to 6 week old mice were lightly sedated with 5% isoflurane (IsoFlo, Abbott Laboratories) in oxygen and 5×10^5^ CFU were pipetted in 50 ul of phosphate-buffered saline (PBS) (Omnipur, Gibbstown, NJ) onto the external nares. This method reliably distributes the bacteria throughout the respiratory tract [43]. To obtain serum, blood from inoculated or vaccinated mice was obtained 28 days post-inoculation and serum was separated from the blood by centrifugation at 500×*g* for 5 minutes.

**Figure 5:**
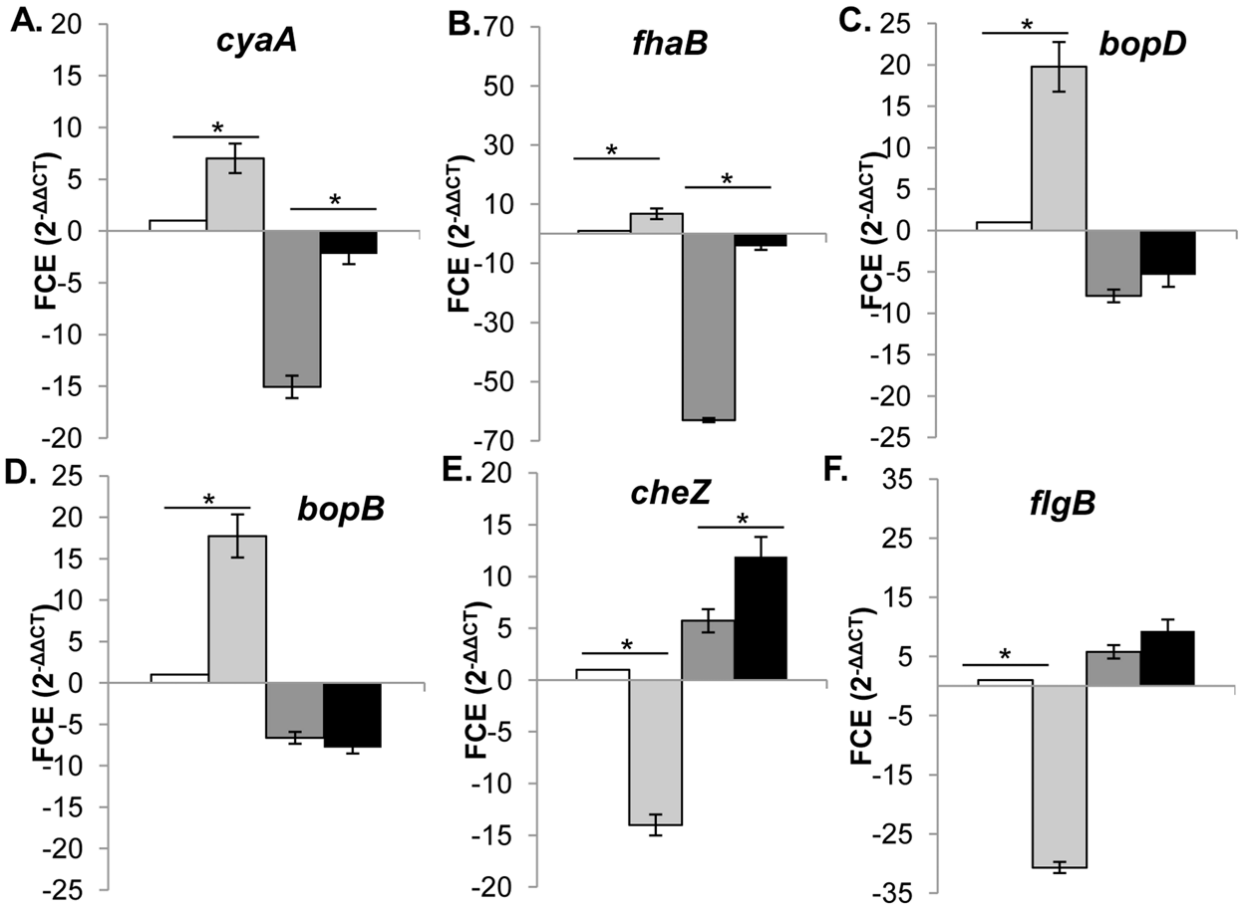
Differential transcription of 5% CO_2_ responsive genes independent of *bvgS* expression. qRT-PCR analysis was performed on RB50 grown in 5% CO_2_ (light grey bars), RB54 in ambient air (dark gray bars), and RB54 in 5% CO_2_ (black bars) compared to RB50 grown in ambient air (white bars). Fold-change expression (FCE) in all strains was expressed as mean ± standard deviation for *cyaA* (A), *fhaB* (B), *bopD* (C), *bopB* (D), *cheZ* (E), and *flgB* (F). Data shown are averages obtained from quadruplicate cultures. * indicates a p-value less than 0.05.

**Figure 6:**
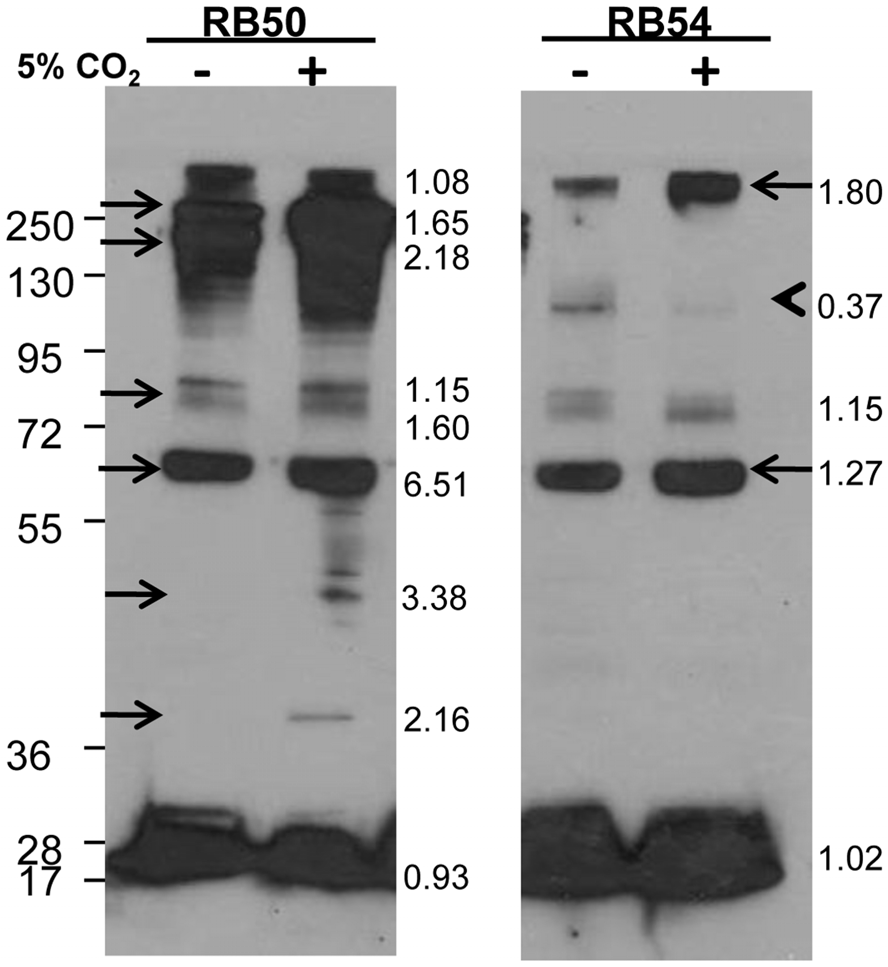
Differential recognition of antigens of a Bvg ^−^ **mutant grown in atmospheric or 5% CO_2_ conditions.** C57BL/6 mice were inoculated with 5×10^5^ CFU *B. bronchiseptica* strain RB50 and sera were collected 28 days post-inoculation. Strains RB50 and RB54 were grown in atmospheric or in 5% CO_2_ concentrations, and were probed with serum against RB50. The ratio of band intensity between antigens produced in ambient air and 5% CO_2_ conditions is indicated in the margins: 1 = equal amounts produced in either conditions, <1 = more produced in ambient air, >1 = more produced in 5% CO_2_ conditions.

### Western Immunoblots

Western blots were performed on whole cell extracts of *B. bronchiseptica*, *B. pertussis* and *B. parapertussis* grown to mid-log phase in SS broth as described previously [38], [39]. Lysates were prepared by resuspending 1×10^9^ CFU in 100 µl of Laemmli sample buffer; total cellular protein content were quantitated using the BCA assay to equalize protein content between samples. 1×10^8^ CFU (10 µl) were run on an 8% sodium dodecyl sulfate-polyacrylamide electrophoresis gels in denaturing conditions and transferred to a polyvinylidene difluoride membrane (Millipore, Bedford, MA). Membranes were probed with pooled serum from mice inoculated with *B. bronchiseptica*, *B. pertussis*, *B. parapertussis*, or a monoclonal antibody against ACT (anti-ACT) at the following dilutions, 1∶1000, 1∶500, 1∶1000 and 1∶1000, respectively. A 1∶10,000 dilution of goat anti-mouse Ig HRP conjugated antibody (Southern Biotech, Birmingham, AL) was used as the detector antibody. Membranes were visualized with ECL Western blotting detection reagents (Amersham Biosciences, Piscataway, NJ) and quantified using Image J software [44].

**Figure 7:**
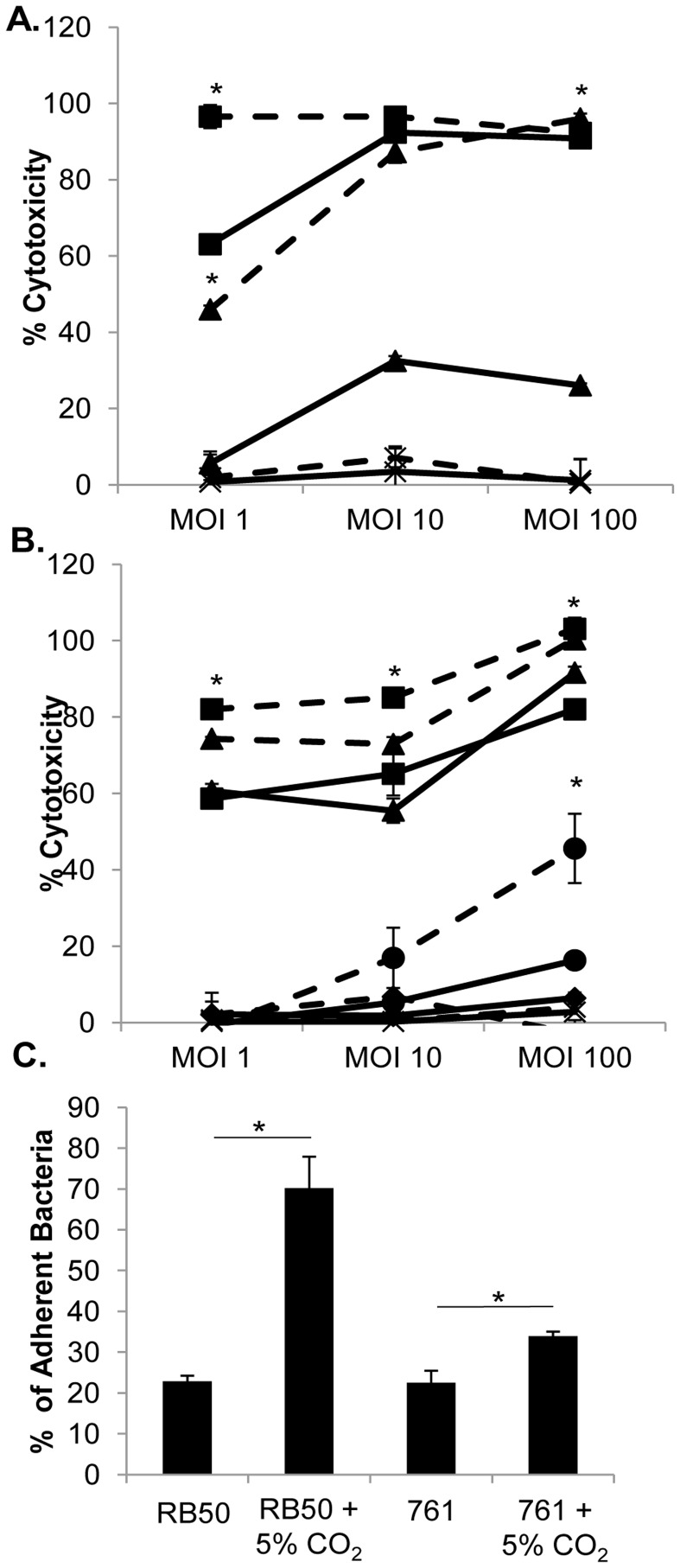
Cytotoxicity and adherence of strains grown in 5% CO_2_ conditions. J774 murine macrophage cells were stimulated with media alone or media (X) containing RB50 (▪) or 761 (▴) (A), RB50 (▪), RB50Δ*cyaA* (▴), RB50Δ*bscN* (•), or RB50Δ*cyaA*Δ*bscN* (♦) (B) in atmospheric (solid lines) or 5% CO_2_ conditions (dashed lines) at MOIs of 1, 10 or 100 for 4 hours, and LDH release was assayed. (C) Rat epithelial cells were incubated with RB50 or 761 at a MOI of 100 for 30 minutes. Adherence is expressed as the proportion of adherent bacteria to the amount in the original inoculum. The error bars represent standard deviations. * p-values less than 0.05 as compared to the same strain grown in atmospheric conditions.

### RNA Isolation

RNA was isolated from three independent biological replicates of *B. bronchiseptica* strains RB50, 761, RB54, 536 and 12822 grown in SS broth overnight. Bacteria were subcultured at a starting OD_600_ of 0.1 into 5 ml of SS broth and grown at 37°C while shaking in either atmospheric or 5% CO_2_ conditions until the OD_600_ reached 0.75. Bacteria were harvested and total RNA was extracted using a RNAeasy Kit (Qiagen, Valencia, CA) and treated with RNase-free DNase I (Invitrogen, Carlsbad, CA) according to the manufacturer’s instructions.

**Figure 8:**
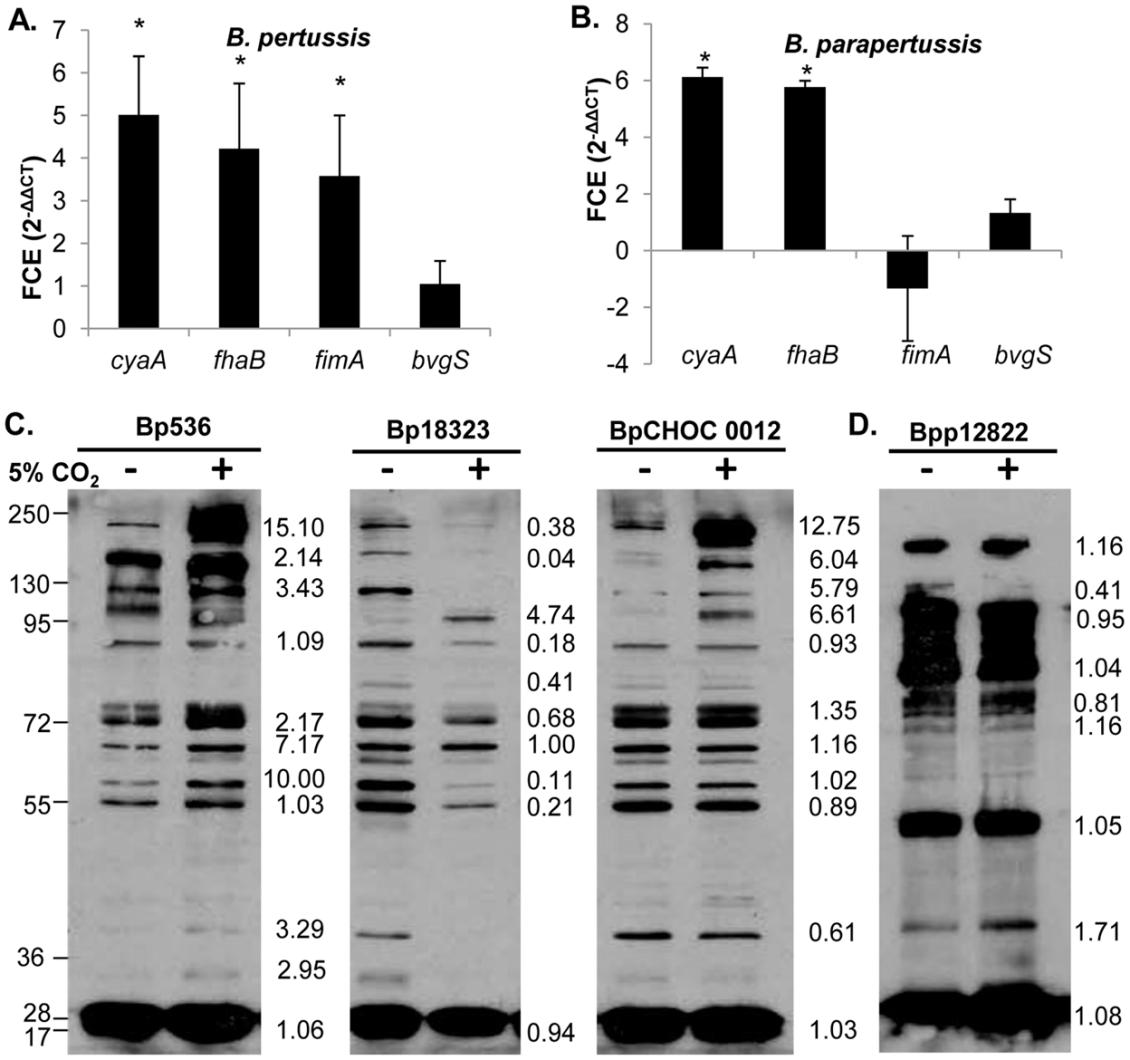
Differential expression of virulence factor genes in *B. pertussis* and *B. parapertussis* in response to 5% CO_2_ conditions. qRT-PCR analysis was performed on *B. pertussis* (A) and *B. parapertussis* (B) grown in atmospheric or elevated CO_2_ conditions. Fold-change expression (FCE) in *B. pertussi*s and *B. parapertussis* grown in 5% CO_2_ was compared to expression in atmospheric levels of CO_2_ and expressed as the mean ± standard deviation. Lysates from bacteria grown in either atmospheric conditions or 5% CO_2_ conditions were probed with serum from mice inoculated with either a *B. pertussis* strain 536 (C) or *B. parapertussis* strain 12822 (D). The ratio of band intensity between antigens produced in ambient air and 5% CO_2_ conditions is indicated in the margins: 1 = equal amounts produced in either conditions, <1 = more produced in ambient air, >1 = more produced in 5% CO_2_ conditions.

### Preparation of Labeled cDNA and Microarray Analysis

RNA isolated from strains RB50 and 761 were used in microarray experiments. A 2-color hybridization format was used for the microarray analysis. For each biological replicate, RNA extracted from cells grown in 5% CO_2_ conditions was used to generate Cy5-labeled cDNA and RNA extracted from cells grown in atmospheric conditions was used to generate Cy3-labeled cDNA. Additionally, dye-swap experiments were performed analogously, in which the fluorescent labels were exchanged to ensure that uneven incorporation did not confound our results. Fluorescently-labeled cDNA copies of the total RNA pool were prepared by direct incorporation of fluorescent nucleotide analogs during a first-strand reverse transcription (RT) reaction [39], [45]–[47]. The two differentially labeled reactions were then combined and directly hybridized to a *B. bronchiseptica* strain RB50-specific long-oligonucleotide microarray [46]. Slides were then scanned using a GenePix 4000B microarray scanner and analyzed with GenePix Pro software (Axon Instruments, Union City, CA). Spots were assessed visually to identify those of low quality and arrays were normalized so that the median of ratio across each array was equal to 1.0. Spots of low quality were identified and were filtered out prior to analysis. Ratio data from the two biological replicates were compiled and normalized based on the total Cy3% intensity and Cy5% intensity to eliminate slide to slide variation. Gene expression data were then normalized to 16S rRNA. The statistical significance of the gene expression changes observed was assessed by using the significant analysis of microarrays (SAM) program [48]. A one-class unpaired SAM analysis using a false discovery rate of 0.001% was performed. Hierarchical clustering of microarray data using Euclidean Distance metrics and Average Linkage clustering was performed using MeV software from TIGR [49]. All microarray data are available in Tables S1 and S2 and have been deposited in ArrayExpress or ArrayExpress Archive under accession number E-MEXP-2875.

### Real-time qPCR (qPCR)

qPCR was performed using a modified protocol previously described [46], [47]. RNA was extracted as described, and 1 µg of RNA from each biological replicate was reverse transcribed using ImProm-II Reverse transcriptase and 0.5 µg of random oligonucleotide hexamers (Promega, Madison, WI). cDNA was diluted 1∶1,000 and 1 µl was used in RT-qPCRs containing 300 nM primers designed with Primer Express software (Applied Biosystems, Foster City, CA, and Integrated DNA Technologies software, www.idtdna.com) (Primer sequences are listed in Table S3) and SYBR Green PCR master mix (Invitrogen, Carlsbad, CA). Samples without reverse transcriptase were included to confirm lack of DNA contamination and dissociation curve analysis was performed to determine cycle threshold (*C_T_*) for each reaction. Amplification of the *recA* RNA amplicon was used as an internal control and for data normalization. Change in transcript level was determined using the relative quantitative method (ΔΔ*C_T_*) [50]. Results were analyzed using analysis of variance with a Tukey simultaneous test, and a *P* value of <0.05 was considered significant.

### Cytotoxicity Assay

Cytotoxicity assays were carried out as previously described [45]. J774A.1 cells, a murine macrophage cell line, obtained from the ATCC were cultured in DMEM with 10% FBS. Cells were grown to approximately 80% confluency, and bacteria were added at a MOI of 100, 10 or 1. After a 5 minute centrifugation at 250×*g*, the mixture was incubated at 37°C for the indicated times. Cytotoxicity was determined by measuring lactate dehydrogenase (LDH) release using the Cytotox96 (Promega, Madison, WI) kit according to the manufacturer’s protocol. Results were analyzed using analysis of variance with a Tukey simultaneous test, and a *P* value of <0.05 was considered significant.

### Adherence Assay

Adherence assays were modified from a previously described protocol [47]. Rat epithelial cell line L2, obtained from the ATCC, was cultured in DMEM/Ham’s F12 50–50 mixture with 10% FBS. Cells were grown to approximately 80% confluency, and bacteria were added at an MOI of 100. After a 5 minute centrifugation at 250×*g*, the mixture was incubated for 30 minutes. Cell culture supernatant was removed and cells were washed 4 times with PBS to remove unbound bacteria. Epithelial cells were then trypsinized and resuspended in 1 mL of tissue culture media. The mixture of cells and bacteria were diluted in PBS and plated on BG agar to determine CFU. Results were analyzed using analysis of variance with a Tukey simultaneous test, and a *P* value of <0.05 was considered significant.

## Results

### A *B. bronchiseptica* Isolate Regulates ACT Expression in Response to 5% CO_2_ Conditions

*B. bronchiseptica* isolates are generally β-hemolytic when grown in Bvg^+^ conditions due to the production of ACT, which causes lysis of red blood cells. It was recently discovered that some *B. bronchiseptica* isolates do not have the genes required to produce a functional ACT and therefore are not hemolytic on blood agar plates [39]. However, through screening of 73 isolates based on hemolysis on blood agar plates and PCR amplification of the genes encoding ACT, 4 *B. bronchiseptica* isolates were found to be non-hemolytic, but still retained the genes for production of ACT (Fig. 1, data not shown). When one isolate displaying this phenotype, *B. bronchiseptica* strain 761, was grown in a tissue culture incubator where the CO_2_ concentration is increased to 5%, it was hemolytic (Fig. 1, compare panels B and D). RB50 was hemolytic even in ambient air (∼0.03% CO_2_) (Fig. 1, compare panels A and C), but there appeared to be more hemolysis when it was grown in 5% CO_2_ conditions, suggesting both strains produce more ACT in response to growth in 5% CO_2_ conditions. Growth rate and pH were not significantly affected by additiona of 5% CO_2_ (Figure S1.), although other indirect effects are possible.

To more directly assess the production of ACT, lysates of strains RB50 and 761 grown in liquid cultures (mid-log phase, O.D. 0.7–0.9) in normal atmospheric conditions or 5% CO_2_ conditions were probed with a monoclonal antibody to the *cyaA* protein product, ACT. RB50 produced more ACT when grown in 5% CO_2_ conditions than in normal atmospheric conditions (Fig. 1E). Strain761 grown in normal atmospheric conditions produced no detectable ACT, while 761 in 5% CO_2_ conditions did produce ACT (Fig. 1E). To determine if strain 761 produces a functional ACT, cyclic-AMP (cAMP) was measured in murine macrophages stimulated with bacteria grown in either normal or 5% CO_2_ conditions. Cells were stimulated for 30 minutes with strains RB50 or 761 to assess their effects on cAMP levels. Both strains grown in 5% CO_2_ induced significantly more cAMP than the same strains grown in normal atmospheric conditions (Fig. 1F, G). Together, these data demonstrate by different measures that the prototypical *B. bronchiseptica* strain, RB50, and strain 761 increase production of functional ACT when grown in 5% CO_2_ conditions, but only strain 761 appears to be dependent on 5% CO_2_ conditions for production of ACT.

Since ACT production was increased in 5% CO_2_, we hypothesized that other antigens might also be differentially regulated in response to these conditions. To test this, Western blot analysis was performed by probing RB50 and 761 lysates, grown in ambient air or in 5% CO_2_ conditions, with serum antibodies from animals convalescent from RB50 infection. No antigens appeared to be produced in greater amounts in RB50 grown in ambient air, while strain 761 produced antigens of between 72 and 95 kDa in greater amounts in 5% CO_2_ growth conditions (Fig. 2, arrowheads). Antigens greater than 130 kDa were produced in greater amounts when RB50 was grown in 5% CO_2_ conditions compared to ambient air (Fig. 2, arrows). Additionally, both 761 and RB50 produced antigens between 36 and 55 kDa in greater amounts when grown in elevated CO_2_ concentrations than in ambient air (Fig. 2). These data indicate that additional antigens besides ACT are differentially regulated in response to 5% CO_2_.

### Defining a CO_2_ Responsive Regulon in *B. bronchiseptica*

To determine which genes are differentially regulated in response to different CO_2_ concentrations, microarray analyses were performed comparing RB50 grown in atmospheric concentrations of CO_2_ to growth in elevated CO_2_ conditions. Transcript abundance of 35 genes increased in RB50 in response to 5% CO_2,_ based on SAM analysis (Table S1, Fig. 3), including genes encoding ACT, as well as genes encoding other known virulence factors such as members of the TTSS locus (*bscE*, *bscF, bopD*), FHA (*fhaC, fhaB, fhaD, fhaA*), fimbriae (*fimA, fim3*), and Prn, (Fig. 3). Expression of 452 genes were decreased when RB50 was grown in 5% CO_2_, many of which are known to be expressed in the Bvg^−^ phase including, *flaA*, *cheW*, *cheB*, *wbmD*, *flgH*, *fliS*, and *cheD* (Fig. 3). A similar trend was observed for strain 761; genes encoding known virulence factors were increased in 5% CO_2_ growth conditions while genes for flagellar assembly and chemotaxis were decreased in these conditions (Table S2, Fig. 3). Expression of 6 genes, including *cyaA,* was increased in both strains and expression of 41 genes decreased in both strains when grown in 5% CO_2_ conditions (Table S4). qPCR of 12 genes confirmed the microarray results (Table S3). Overall, these data indicate there is a CO_2_ responsive regulon in *B. bronchiseptica* that includes several virulence factors, suggesting a role during infection.

Among the 35 genes in strain RB50 increased in expression in 5% CO_2_ conditions, 19 genes were reported to be positively regulated under Bvg^+^ conditions, 4 genes negatively regulated by BvgAS, and 13 genes not previously known to be regulated by BvgAS, based on previous analysis [46], [51]. Similarly, of the 452 genes negatively regulated by 5% CO_2_, 252 were known to be negatively regulated under Bvg^+^ conditions, 19 were positively regulated and 181 were not previously known to be regulated by BvgAS. The CO_2_-responsive regulon appears to contain genes that are Bvg-regulated, as well as genes that are not, suggesting a regulatory mechanism that functions independently or cooperatively with BvgAS rather than subordinate to it.

### CO_2_ Responsiveness is Not Conferred by Differences in the *bvgAS* Loci between Strains

Three additional *B. bronchiseptica* strains, JC100, 308 and 448, were also observed to be hemolytic only when grown in 5% CO_2,_ but not in normal atmospheric conditions. Changes in virulence factor expression have previously been attributed to variation in *bvgAS* and since some CO_2_-responsive genes are Bvg-regulated, the *bvg* locus of these strains was analyzed revealing that strain JC100 carries a 29 amino acid duplication in the region of the *bvgS* gene encoding the periplasmic domain (Fig. 4). To determine if this duplication is involved in the CO_2_/ACT dependent phenotype in JC100, the *bvgAS* locus from JC100 was expressed in a RB50 knockout of *bvgAS* (RB55::pBvgAS_JC100_). This strain was hemolytic in the absence of 5% CO_2_, indicating that transfer of the *bvgAS* locus does not confer the CO_2_-dependence for ACT production (Table S5). The reverse was also true; when a plasmid carrying the *bvgAS* locus from RB50 was introduced into JC100 (MLJC114::pEG100), ACT production remained dependent on growth in 5% CO_2_ (Table S5). Furthermore, the *bvgS* gene of 761 did not have this duplication (Fig. 4). These data indicate that the duplication in *bvgS* in JC100 is neither necessary nor sufficient for the CO_2_ requirement for hemolysis.

### CO_2_ Responsiveness in the *B. bronchiseptica* Bvg^−^ State

Since some virulence genes known to be Bvg-regulated were responsive to 5% CO_2_ conditions, we sought to determine if they are differentially regulated in response to 5% CO_2_ in the absence of BvgAS. Transcription of six genes responsive to CO_2_, *cyaA*, *fhaB, bopD, bopB, cheZ* and *flgB*, were analyzed in *B. bronchiseptica* RB50 and RB54, a Bvg^–^phase locked derivative of strain RB50, grown in normal atmospheric or 5% CO_2_ conditions (Fig. 5). For RB50, addition of 5% CO_2_ increased transcription of *cyaA*, *fhaB*, *bopD* and *bopB* (Fig. 5A–D), but decreased transcription of *cheZ* and *flgB* (Fig. 5 E,F). In RB54, transcription of *bopD* and *bopB* was not increased in response to addition of 5% CO_2_ (Fig. 5C,D), and transcription of *cheZ* and *flgB* was not decreased (Fig. 5 E,F). Therefore, the differential transcription of *bopD*, *bopB*, *cheZ* and *flgB* in response to 5% CO_2_ is dependent on BvgS. However, in the *bvgS* mutant RB54, the transcription of genes *cyaA* and *fhaB* was increased in response to addition of 5% CO_2_ (Fig. 5 A,B), indicating that some gene regulation in response to 5% CO_2_ is independent of BvgS.

To determine whether differential transcription results in differential accumulation of antigens in the absence of BvgS, Western blots were performed. Lysates from RB54 grown in normal atmospheric or 5% CO_2_ conditions were probed with serum antibodies from mice convalescent from RB50 infection (Fig. 6). Strain RB54 grown in 5% CO_2_ also produced antigens >250 kDa and ∼60 kDa in greater amounts (Fig. 6). RB54 grown in ambient air produced an antigen between 95 and 130 kDa in greater amounts (Fig. 6, arrowhead). These data demonstrate that antigen production is differentially regulated in response to 5% CO_2_ even when a functional BvgS is absent.

### Growth in 5% CO_2_ Affects Cytotoxicity and Adherence of *B. bronchiseptica* Strains

Since genes, *cyaA* and the TTSS genes, associated with the cytotoxicity of *B. bronchiseptica* were increased when strains RB50 and 761 were grown in 5% CO_2_ conditions (Fig. 3), we assessed the relative cytotoxicity to J774 murine macrophages of strains grown in atmospheric or 5% CO_2_ conditions. Similar to previous findings [52], RB50 killed >90% of cells at an MOI of 10 or 100; however, RB50 only killed ∼65% at an MOI of 1 (Fig. 7A). RB50 grown in 5% CO_2_ killed >90% at all MOIs, indicating that RB50 grown in 5% CO_2_ killed more macrophages at a lower MOI than RB50 grown in atmospheric conditions (Fig. 7A). Strain 761 had detectable (∼30%) killing only at high MOIs (10 and 100), while 761 grown in 5% CO_2_ was cytotoxic at an MOI of 1 (∼45%) and comparable to RB50 at higher MOIs (Fig. 7A). These data show that growth in 5% CO_2_ increased killing of murine macrophages by both strains.

ACT and TTSS have been previously shown to account for all cytotoxicity of macrophages when RB50 is grown in ambient air [37], [52]. Since 5% CO_2_ increased expression of several genes, we examined whether the increased cytotoxicity is due to increases in these known factors or a new cytotoxic mechanism. Cells were exposed for 4 hours at MOIs of 1, 10 or 100 with wild-type RB50, RB50*ΔcyaA,* RB50Δ*bscN* (encoding the ATPase of the TTSS) or a mutant lacking *bscN* and *cyaA* (RB50Δ*cyaA*Δ*bscN*). Growth in 5% CO_2_ increased cytotoxicity of RB50Δ*cya* to macrophages at MOIs of 1, 10 and 100, while growth 5% CO_2_ caused increased cytotoxicity of RB50Δ*bscN* only at an MOI of 100, likely indicating the differential roles of the TTSS and ACT in cytotoxicity (Figure 7B). RB50Δ*cyaA*Δ*bscN* caused very low levels of cytotoxicity as observed previously [37], and growth in 5% CO_2_ did not increase cytotoxicity(Fig. 7B). These data suggest that there are no other cytotoxic mechanisms and that increased ACT and TTSS function accounts for the increased cytotoxicity when strains are grown in 5% CO_2_ conditions.

Since many genes encoding adhesins were increased in transcription in response to growth in 5% CO_2_ conditions, we hypothesized that strains grown under these conditions, in comparison to growth in ambient air, would be more adherent to epithelial cells. L2 cells were incubated with RB50 or 761 pre-grown in either atmospheric or 5% CO_2_ conditions. Both strains pre-grown in 5% CO_2_ conditions adhered to lung epithelial cells more efficiently than bacteria grown in normal atmospheric conditions (Fig. 7C).

### *B. pertussis* and *B. parapertussis* Modulate Virulence Factor Expression in Response to 5% CO_2_

Since up-regulation of virulence factors in response to growth in 5% CO_2_ conditions is common to multiple *B. bronchiseptica* strains, we hypothesized that *B. pertussis* and *B. parapertussis* may also regulate virulence factor expression in response to growth in 5% CO_2_ conditions. Genes shown to be CO_2_ responsive (*cyaA, fhaB, fimA*) or non-responsive to CO_2_ (*bvgS*) in *B. bronchiseptica* (Fig. 3), were chosen to be analyzed by qPCR in *B. pertussis* and *B. parapertussis*. *B. pertussis* and *B. parapertussis* had increased expression of *fhaB* and *cyaA*, but not *bvgS* in response to CO_2_ (Fig. 8A, B). *B. pertussis*, unlike *B. parapertussis*, also had increased expression of *fimA*, indicating that the 5% CO_2_ responsive regulon may be different among the three classical *Bordetella* species. To further investigate this effect, Western blots with lysates of *B. pertussis* and *B. parapertussis* grown in ambient air or 5% CO_2_ were probed with either *B. pertussis-*induced sera or *B. parapertussis*-induced sera (Fig. 8C, D). *B. pertussis* strains 536, 18323 and a recent clinical isolate CHOC 0012 grown in ambient air showed a different antigenic profile from the lysates prepared from strains grown in 5% CO_2_, with bands from roughly 55 to 250 kDa which were more numerous and intense in 5% CO_2_ for strains 536 and CHOC 0012 (Fig. 8C). Notably, *B. pertussis* strains 536 and CHOC 0012 appeared to increase similar antigens in response to growth in 5% CO_2_ conditions, while strain 18323 grown in 5% CO_2_ decreased production of several antigens (Figure 8C). *B. parapertussis* grown in ambient air produced a more intense band between 130 and 250 kDa, while growth in 5% CO_2_ produced a more intense band between 36 and 55 kDa (Fig. 8D). Overall, *B. parapertussis* did not appear to differentially regulate many antigens in response to growth in 5% CO_2_ conditions (Figure 8D). These data indicate that several antigens in *B. pertussis*, but few in *B. parapertussis* isolate 12822, are differentially regulated in response to growth in 5% CO_2_ compared to growth in ambient air and that there is strain variation in CO_2_ responsiveness in the bordetellae.

## Discussion

BvgAS was originally considered an ON/OFF switch, modulating *Bordetella* species between two distinct states, avirulent (Bvg^−^) and virulent (Bvg^+^). The discovery of an intermediate phase has led to a view of BvgAS gene regulation as a rheostat visualized as varying along a one dimensional gradient [23], [32], [34]. In this view of the two-component system few signals, temperature and some chemical cues, are known to affect virulence factor regulation through the BvgAS system. However, the respiratory tract contains many microenvironments, and within each environment there is likely to be great variation. For example, CO_2_ levels are thought to vary between air and epithelial cells of the respiratory tract, although these are separated by a fraction of a millimeter of mucous. These sites also change dramatically in the course of the various stages of an infection, and there is likely to be a selective advantage to any strain that can sense these differences and modulate virulence factor expression in response.

Here we show that the classical bordetellae share the ability to sense and respond to physiological changes in CO_2_ concentrations likely to be encountered in the host. In mammalian tissues and blood, CO_2_ concentrations are higher than inhaled ambient air concentrations of CO_2_, which are approximately 0.03%. The observed changes in expression of various virulence factors (Fig. 3), and altered phenotypes (adherence and cytotoxicity, Fig. 7) provide additional evidence that the ability to respond to changes in CO_2_ concentrations allow *Bordetella* species to adjust to different microenvironments within the host respiratory tract.

Recently, it has been shown that there is a zone of oxygenation between the anaerobic luminal environment and the host epithelium in the gastrointestinal tract, which can be sensed by *Shigella flexneri* [2]. The presence of oxygen alters the expression of TTSS effectors that are important for invasion of host cells, and this ‘aerobic zone’ may enhance secretion of these effectors thereby increasing invasion of host cells [2]. Similarly, the respiratory tract of mammals contains multiple sites where gradients of CO_2_ or oxygen likely influence virulence factor expression and how respiratory pathogens interact with host cells. *B. bronchiseptica* has been isolated from multiple sites within the respiratory tract (e.g. nasopharnyx, trachea, lungs) and the ability to detect these differences could allow this pathogen to respond by expressing the array of factors optimal for success under each condition [2]. As bacteria disseminate from the nasal cavity to the trachea, lung and potentially even invade tissues, CO_2_ concentrations may increase, serving as a signal for increased transcription of factors such as adhesinsand toxins that subvert the immune response, which is more robust in these regions 17,52–54.

*B. bronchiseptica* strains sense and differentially regulate virulence factor gene expression in response to 5% CO_2_ (Fig. 3), and differential regulation was observed in multiple strains and species of *Bordetella* demonstrating that sensing and responding to carbon dioxide levels is an ability shared among the classical bordetellae. Additionally, the transcription of several Bvg^+^-phase genes increased in response to 5% CO_2_, suggestive of BvgAS involvement in the response. Interestingly, regulation of some virulence factor genes (*bopD*, *bopB*) by BvgAS was epistatic to 5% CO_2_ regulation. However, not all virulence gene expression (*cyaA*, *fhaB*) was dependent on *bvgS* (Fig. 5A, B), demonstrating an independent mechanism for virulence factor gene regulation in response to 5% CO_2_.

Standard Bvg^+^ conditions, without additional CO_2_, are sufficient for production of ACT (Fig. 1) in RB50, suggesting that additional mechanisms may contribute to increases in production, but are not required. Of 73 *B. bronchiseptica* strains screened, 4 strains were identified here, 761, 308, 448 and JC100, in which Bvg^+^ phase conditions are not sufficient for measurable production of ACT. In these strains both Bvg^+^ phase conditions and 5% CO_2_ are required for detectable production of ACT. The requirement for 5% CO_2_ for the production of virulence factors may reflect evolutionary adaption of *B. bronchiseptica* strains, and suggests that the mechanism of CO_2_ sensing may confer a selective advantage. Intriguingly, there also appears to be variation in responsiveness of both *B. bronchiseptica* and *B. pertussis* strains suggesting that although the ability to respond appears to be conserved the regulon may vary between species and strains.

Collectively these data demonstrate that a CO_2_ response mechanism contributes to regulation of virulence factors in the classical bordetellae (Fig. 5A, B). This is the first description of a CO_2_ sensing mechanism that regulates virulence factor expression cooperatively with, or independently of, BvgAS. Our data support the idea that virulence factor gene expression can be fine-tuned in response to signals specific to different microenvironments within the respiratory tract or deeper tissues within the host.

## Supporting Information

Figure S1 **Growth and pH of** ***B. bronchiseptica*** **strains RB50 and 761 in ambient air or 5% CO_2_ conditions.** The growth of strains RB50 (diamonds) and 761(squares) grown in normal atmospheric oxygen conditions (black) or in elevated 5% CO_2_ conditions (white) was measured. pH was assessed at the indicated timepoints expressed as the mean ± standard deviation.
(PDF)

Table S1 *B. bronchiseptica* strain RB50 Expression Arrays 1 & 2.
(XLS)

Table S2 *B. bronchiseptica* strain 761 Expression Arrays 1 & 2.
(XLSX)

Table S3 qRT-PCR Data and Primers.
(XLSX)

Table S4 Genes Increased and Decreased in Transcription in response to 5% CO_2_ conditions in *B. bronchiseptica* strains RB50 and 761.
(XLSX)

Table S5 Colony characteristics of RB50 and JC100 derivatives under different conditions.
(XLSX)
