# Supplementary figures and images for: Identification of a CO2 Responsive Regulon in Bordetella
Source: PLoS One. 2012 Oct 24;7(10):e47635. doi: 10.1371/journal.pone.0047635 (PMC3480411; doi:10.1371/journal.pone.0047635)

**Figure S1.**

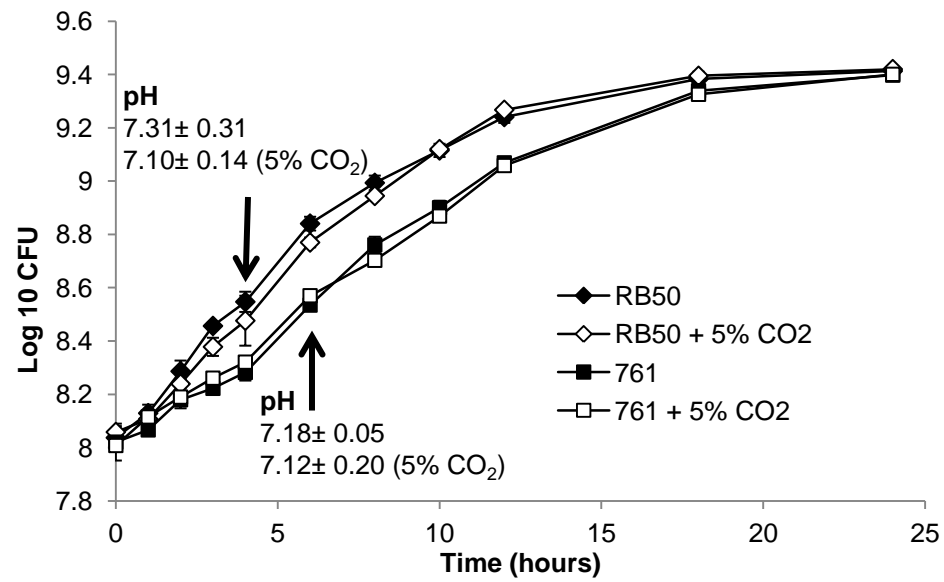

Supplement: Figure S1 — Growth and pH of B. bronchiseptica strains RB50 and 761 in ambient air or 5% CO2 conditions. The growth of strains RB50 (diamonds) and 761(squares) grown in normal atmospheric oxygen conditions (black) or in elevated 5% CO2 conditions (white) was measured. pH was assessed at the indicated timepoints expressed as the mean ± standard deviation. (PDF) [file pone.0047635.s001.pdf]
